# Senescence-Inflammatory Regulation of Reparative Cellular Reprogramming in Aging and Cancer

**DOI:** 10.3389/fcell.2017.00049

**Published:** 2017-05-05

**Authors:** Javier A. Menendez, Tomás Alarcón

**Affiliations:** ^1^Metabolism and Cancer Group, Program Against Cancer Therapeutic Resistance, Catalan Institute of OncologyGirona, Spain; ^2^Molecular Oncology Group, Girona Biomedical Research Institute (IDIBGI)Girona, Spain; ^3^METABOSTEMBarcelona, Spain; ^4^Institució Catalana de Recerca i Estudis Avançats (ICREA)Barcelona, Spain; ^5^Computational and Mathematical Biology Research Group, Centre de Recerca MatemàticaBarcelona, Spain; ^6^Departament de Matemàtiques, Universitat Autònoma de BarcelonaBarcelona, Spain; ^7^Barcelona Graduate School of MathematicsBarcelona, Spain

**Keywords:** reprogramming, aging, cancer, senescence, inflammation

## Abstract

The inability of adult tissues to transitorily generate cells with functional stem cell-like properties is a major obstacle to tissue self-repair. Nuclear reprogramming-like phenomena that induce a transient acquisition of epigenetic plasticity and phenotype malleability may constitute a reparative route through which human tissues respond to injury, stress, and disease. However, tissue rejuvenation should involve not only the transient epigenetic reprogramming of differentiated cells, but also the committed re-acquisition of the original or alternative committed cell fate. Chronic or unrestrained epigenetic plasticity would drive aging phenotypes by impairing the repair or the replacement of damaged cells; such uncontrolled phenomena of *in vivo* reprogramming might also generate cancer-like cellular states. We herein propose that the ability of senescence-associated inflammatory signaling to regulate *in vivo* reprogramming cycles of tissue repair outlines a threshold model of aging and cancer. The degree of senescence/inflammation-associated deviation from the homeostatic state may delineate a type of thresholding algorithm distinguishing beneficial from deleterious effects of *in vivo* reprogramming. First, transient activation of NF-κB-related innate immunity and senescence-associated inflammatory components (e.g., IL-6) might facilitate reparative cellular reprogramming in response to acute inflammatory events. Second, para-inflammation switches might promote long-lasting but reversible refractoriness to reparative cellular reprogramming. Third, chronic senescence-associated inflammatory signaling might lock cells in highly plastic epigenetic states disabled for reparative differentiation. The consideration of a cellular reprogramming-centered view of epigenetic plasticity as a fundamental element of a tissue's capacity to undergo successful repair, aging degeneration or malignant transformation should provide challenging stochastic insights into the current deterministic genetic paradigm for most chronic diseases, thereby increasing the spectrum of therapeutic approaches for physiological aging and cancer.

The epistemic interest of induced pluripotent stem cells (iPSCs) to model aging and aging-related diseases largely relies on the appreciation of nuclear reprogramming as a disease-in-a-dish technology. Expression of the Yamanaka cocktail of transcription factors (i.e., *Oct4, Sox2, Klf4*, and *c-Myc*, OSKM; Takahashi and Yamanaka, 2006; Takahashi et al., 2007) is commonly viewed as an artificial, non-naturally occurring molecular modality capable of radically modifying the cellular identity of differentiated cells *in vitro* (Liu et al., 2012; Inoue et al., 2014; Sterneckert et al., 2014; Avior et al., 2016). An overlooked dimension of OSKM-driven cellular reprogramming is the potential existence of such a phenomenon as a natural process for *in vivo* tissue rejuvenation. Activation of adult stem/progenitor cells and proliferation of remaining differentiated cells are well-established mechanisms for the replacement of lost cells following injury. A physiological version of OSKM-induced reprogramming might operate as an evolutionary conserved, *bona fide* epigenetic strategy to provide self-repair and resistance to damage and disease (Cooke et al., 2014; Jessen et al., 2015; de Keizer, 2017).

There is growing support for the hypothesis that nuclear reprogramming-like phenomena inducing the short-term acquisition of epigenetic plasticity, followed by cell differentiation and replacement of damaged cells, might be a reparative route through which tissues respond to injuries and other adversities. We herein delineate a threshold model of aging and cancer based on the intercommunication between cellular reprogramming/differentiation cycles of tissue repair and the cell-autonomous and non-cell autonomous mechanisms that initiate and propagate senescence-associated inflammatory signaling.

## Reparative cellular reprogramming *in vivo*: the evidence bases

### Nuclear reprogramming and activation of innate immunity

**Figure 1:**
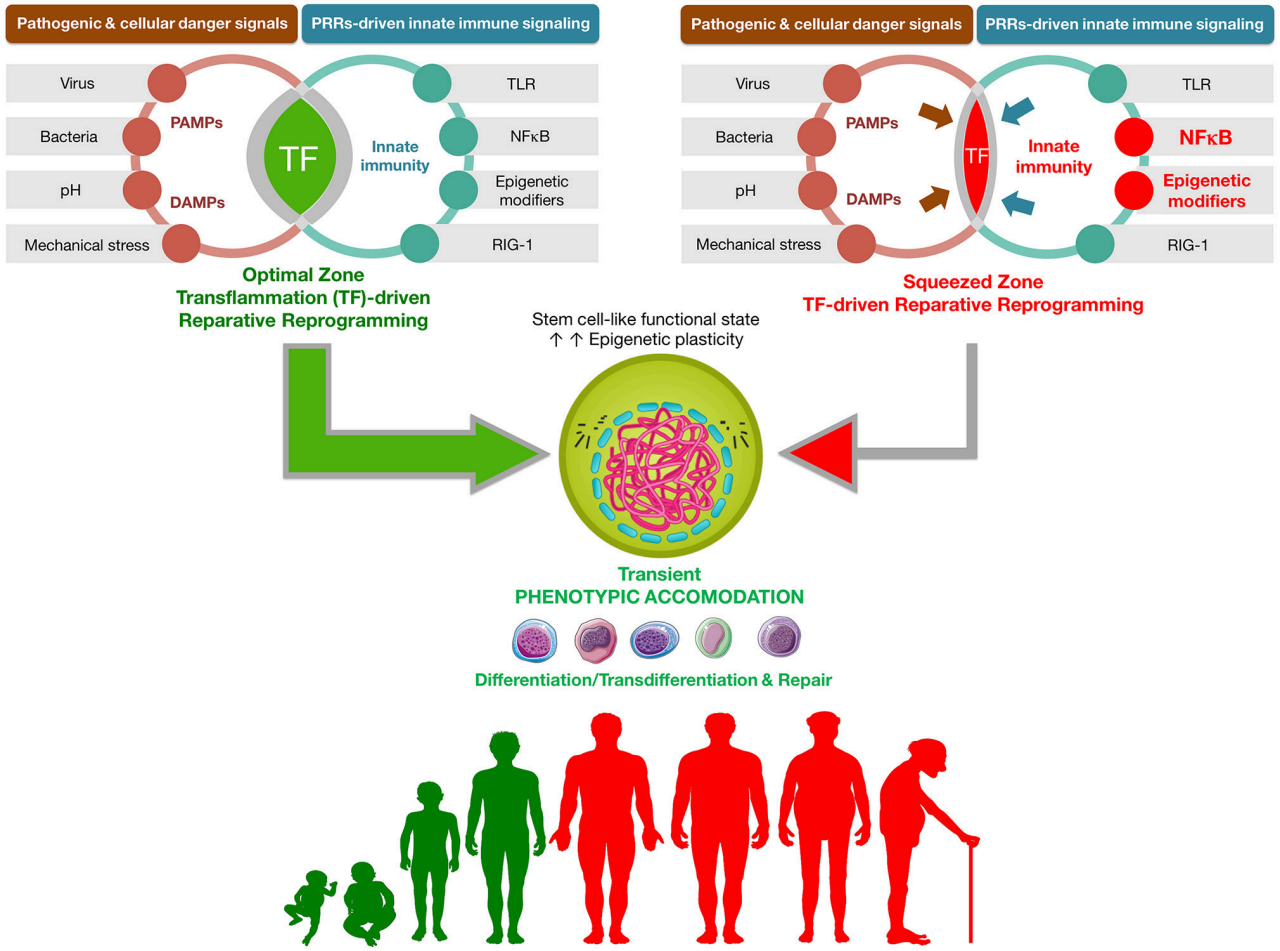
**Transflammation-driven epigenetic plasticity: a paradigmatic example of ***in vivo*** reparative reprogramming**. Transient activation of the PAMPs-DAMPs → NFκB signaling axis may delineate an optimal zone of transflammation (TF)-driven reparative reprogramming characterized by increased epigenetic plasticity and phenotypic malleability capable of responding and adapting to injury, stress, and disease (Lee et al., 2012; O'Neill, 2012; Cooke et al., 2014). The efficiency of NFκB signaling and the level of inflammatory responses is the nodal point linking the pathogenic assault and cellular danger signals and the organization of cellular resistance and tissue repair. NFκB hyperfunction and its interaction with epigenetic modifiers would significantly squeeze the optimal zone of TF-driven reparative reprogramming, thus impairing the adequate organization of defense mechanisms. By operating as the perpetrator of inflammaging, the NFκB signaling integrates the intracellular regulation of transflammation immune responses in both aging and aging-related diseases (Salminen et al., 2008; Montgomery and Shaw, 2015).

The mere process of viral transduction that is frequently employed to deliver OSKM factors into target cells (i.e., the viral particles themselves) can elicit the expression of genes involved in innate immunity (Lee et al., 2012; O'Neill, 2012). Furthermore, only after the efficient activation of innate immunity, considered the phylogenetically oldest mechanism of defense against microbes, can the retrovirally-delivered OSKM factors successfully accomplish cellular reprogramming (Lee et al., 2012; O'Neill, 2012). Key players of innate immunity-signaling, including toll-like receptors (TLRs) such as TLR3 and the retinoic acid-inducible gene 1 receptor (RIG-1)-like receptor (RLR), appear to be necessarily involved in the nuclear reprogramming process to pluripotency (Cooke et al., 2014). Moreover, the retrovirally-induced activation of pattern recognition receptors (PRRs) such as TLRs and RIG-1, which are specialized DNA sensors charged with cell defense *via* sensing nucleic acids generally derived from microbes (e.g., viral RNA) (Broz and Monack, 2011; Newton and Dixit, 2012; Dixit and Kagan, 2013), has been found to stimulate pro-inflammatory NFκB signaling as part of the reprogramming process. Retrovirally-induced immune activation and NFκB-mediated cellular inflammation can trigger significant downstream epigenetic modifications, including decreased H3K9 methylation (indicative of gene silencing) and increased H3K4 methylation (indicative of open chromatin) of the endogenous *Oct4* and *Sox2* gene promoters, thereby facilitating nuclear reprogramming upon delivery of the stemness transcription factors (Lee et al., 2012; O'Neill, 2012). Indeed, this activation of inflammatory signaling appears to autonomously promote epigenetic plasticity by eliciting global changes in the expression and activity of several chromatin-modifying enzymes, such as upregulation of histone acetyltransferases, downregulation of histone deacetylases, and downregulation of histone methyltransferases such as DOT1L (Lee et al., 2012; O'Neill, 2012; Cooke et al., 2014; Figure 1).

### Inflammation and epigenetic plasticity

The targeted modulation of epigenetic modifiers that operate as barriers to OSKM-driven reprogramming is sufficient to more efficiently generate iPSCs with fewer exogenous stemness transcription factors (Onder et al., 2012; Luo et al., 2013; Rais et al., 2013; Soria-Valles et al., 2015a,b). It thus seems reasonable to suggest that the Yamanaka cocktail simply drives induced pluripotency because they efficiently and specifically finalize the epigenetic modification of chromatin pre-initiated as part of the retrovirus-induced PRR-driven host genetic response (Lee et al., 2012; O'Neill, 2012; Cooke et al., 2014). Considering that innate immunity functions not only for the early prevention, control, or elimination of host infection, but also to warn against infection or DNA damage, to which an adaptive immune response has to be mounted, other NFκB-related pro-inflammatory damage receptors (e.g., TLR4 binding to endotoxins such as lipopolysaccharide) may likely act as epigenetic modifiers to promote more epigenetically plastic cellular states (Erdoğan et al., 2016).

In agreement with the host defense nature of inflammation-regulated reprogramming, the Yamanaka factors have been found to cooperate with soluble and contact-dependent stromal signals to accelerate the conversion of myeloid progenitors to a stable pluripotent state (Park et al., 2012). Interestingly, such extrinsic potentiation of the reprogramming capacity of somatic progenitors has been found to involve both an epigenetically permissive genome and the molecular activation of the TLR/NFκB signaling pathway (Park et al., 2012). However, a close relationship seems to exist between an optimal level of cell-autonomous inflammation and the acquisition of cell states epigenetically poised to rapidly provide phenotypic responses to environmental stresses. A landmark study from the Lopez-Otin group revealed that chronic hyperactivation of pro-inflammatory NF-κB signaling constitutes a critical impediment to nuclear reprogramming in both normal and accelerated aging (Soria-Valles et al., 2015a,b). Accordingly, hyperactivation of the histone H3 methyltransferase DOT1L, a central component of the epigenetic program that is down-regulated during retrovirally-induced activation of innate immune/NFκB inflammatory signaling to promote epigenetic plasticity (Lee et al., 2012; O'Neill, 2012; Cooke et al., 2014), was found to conversely operate as an epigenetic barrier causally involved in the loss of tissue plasticity following chronic NF-κB hyperactivation (Soria-Valles et al., 2015a,b; Figure 1).

### Innate immunity and transflammation-thresholded cellular reprogramming

The capacity for pathogen-associated molecular patterns (PAMPs), for example, viruses and bacteria, or damage-associated molecular patterns (DAMPs), such as mechanical stresses, pH, and oxidants, to support OSKM-driven reprogramming could have implications beyond the mere molecular insights into the cell-autonomous mechanistic barriers determining the *in-a-dish* efficiency of nuclear reprogramming. The ability of the inflammatory response to mitigate infection and clear damaged cells seems to be an evolutionary conserved process from lower organisms to mammals that might also function to promote initiation of damage repair and tissue regeneration (Karin and Clevers, 2016). When adult tissue cells are confronted with PAMPs/DAMPs, the PRR-triggered activation of downstream innate immune signaling pathways mobilizes archetypal inflammatory pathways (e.g., NF-κB, interferon regulatory factor-3) to promote an open configuration of the chromatin and, therefore, generate greater epigenetic plasticity. This phenomenon of so-called “transflammation” (Lee et al., 2012; O'Neill, 2012; Cooke et al., 2014) may act to fine-tune the rapid, but transient, adaptive adjustments to the fluidity of the cell phenotype by providing a more plastic epigenetic state for the self-repair of damaged/diseased tissues. Beyond an optimal threshold zone of optimally and transiently activated transflammation, a successful augmentation of epigenetic and phenotypic tissue plasticity would be minimal or totally absent despite damage/disease-driven activation of cellular reprogramming-like processes (Figure 1). Reaching into an optimal zone of transflammation-initiated cellular reprogramming-like phenomena, followed by re-acquisition of the original or alternative cell fate, might allow tissue repair *via* replenishment or transdifferentiation of the original damaged/lost cells (Lee et al., 2012; O'Neill, 2012; Cooke et al., 2014, 2015; Sayed et al., 2015). Conversely, a chronic, cell-autonomous hyperactivation of comparable inflammation-epigenetic axes (e.g., NF-κB), rather than establishing stem-like epigenetic states, will position the damaged/stressed cell outside the optimal zone for cellular reprogramming, impeding tissue rejuvenation and generating an aging phenotype (Figure 1).

### Permissiveness of the *in vivo* environment for nuclear reprogramming-like phenomena

Before unequivocally suggesting that activation of reprogramming *in vivo* can be considered a host genetic program for resistance to disease and damage, it should be clarified whether the *in vivo* conditions are permissive for nuclear programming-like phenomena. By considering that cell-cell fusion is a physiological mechanism controlling not only fertilization, but also developmental processes, and that these events increase following injury and inflammation, Cosma and colleagues investigated whether stem and progenitor cells could fuse with retinal neurons and Müller glia after their transplantation into damaged retinas, and whether the *in vivo*-formed hybrids underwent nuclear reprogramming (Sanges et al., 2013). Using the eye as a model system that has low immune responses to cells and viral vectors, the authors demonstrated that, upon N-methyl-D-aspartate-induced retinal damage, mouse retinal neurons could be transiently reprogrammed back to a precursor stage. This pioneering study was the first to demonstrate that cell-fusion-mediated nuclear reprogramming of terminally differentiated cells should be viewed as a *bona fide* repair mechanism to stimulate cell and tissue regeneration in mammals *in vivo*. Soon after, a landmark study by the Serrano group established the possibility of *in vivo* nuclear reprogramming within tissues (Abad et al., 2013). These authors showed that induction of the OSKM factors in mice promoted not only the emergence of groups of dedifferentiated cells expressing the pluripotency marker *Nanog* in multiple organs, but also the generation of circulating *in vivo* iPSCs with a highly plastic, more primitive totipotent state than embryonic stem cells (ESCs) and *in vitro*-derived iPSCs (Abad et al., 2013). Permissiveness of the *in vivo* environment to reprogramming-like phenomena has been positively supported by two recent breakthrough studies from the Serrano and Izpisua-Belmonte groups; the former showing that tissue damage provides critical signals for OSKM-driven cellular reprogramming *in vivo* (Mosteiro et al., 2016), and the latter revealing how the cyclic, short-term expression of OSKM factors *in vivo* ameliorates cellular and physiological hallmarks of aging (Ocampo et al., 2016).

### Nuclear reprogramming-induced tumorigenesis

Using a murine system in which the expression of reprogramming factors was controlled temporally with doxycycline, the Yamanaka group demonstrated that whereas acute activation of OSKM factors leads to the formation of dysplastic lesions that spontaneously reverse upon doxycycline withdrawal, chronic induction results in the formation not only of well-differentiated teratomas but also of tumor-like undifferentiated tissues unresponsive to doxycycline withdrawal (Hobbs and Polo, 2014; Ohnishi et al., 2014a,b). The manifestation of tumorigenesis in the context of *in vivo* nuclear reprogramming might merely reflect the shared roles of transcription factors and chromatin regulators in mediating cell state transitions, which correspondingly occur during induced pluripotency and during the conversion of differentiated cells into a tumorigenic state (Suvà et al., 2013; Tung and Knoepfler, 2015). However, while gene methylation was found to be significantly perturbed in the so-called partially reprogrammed transformed cells (PRTCs), and the genomic imprints of PRTCs appeared unstable in the absence of permanent genetic aberrations, it should be noted that such epigenetic features were distinguishable from those in sporadic carcinomas. Thus, whereas DNA hypermethylation at proximal promoter regions was not evident, global DNA methylation levels were comparable with those of normal cells, indicating a lack of both site-specific DNA hypermethylation and global DNA hypomethylation that characterizes most human carcinomas (Ohnishi et al., 2014a,b). Cellular reprogramming-associated generation of undifferentiated dysplastic cells in various tissues notably resembled those of Wilms' tumors, the most common pediatric kidney cancer, as well as those of pediatric blastomas such as hepatoblastomas and pancreatoblastomas. These findings support the notion that deleterious nuclear reprogramming-associated epigenetic reorganization in certain organs and tissues at discrete developmental stages can contribute to the initiation and progression of pediatric tumors.

### Pathological versions of nuclear reprogramming

The occurrence of PRTCs does not in itself provide sufficient evidence that de-differentiation is involved in cancer development. The number and complexity of the molecular events required for *de novo* generation of stem cell-like cells (e.g., chromatin decondensation, loss of differentiation marks, transcriptional activation of stemness genes, suppression of competing cell lineages factors, among others) is considered to intrinsically prevent the initiation of pathological versions of nuclear reprogramming-like processes in differentiated tissues, including those of tumors (Pasque et al., 2010, 2011; Cantone and Fisher, 2013; Brooks et al., 2015). Along this line, OSKM-derived PRTCs have been viewed as mechanistically irrelevant for most common sporadic cancers that afflict the elderly, where developmental biology is not commonly considered. Nevertheless, *in vivo* nuclear reprogramming-related PRTCs might form the basis of a new model of epigenetic tumorigenesis when looked at in depth.

Upon reactivation of OSKM factors, PRTCs fully reprogram into iPSCs, suggesting that the reorganization of epigenetic landscapes associated with chronic, unresolved nuclear reprogramming is adequate to generate epigenetically heritable cancer-like phenotypes. If pathological nuclear reprogramming reflects a cancer initiation phenomenon driven purely by epigenetic mechanisms (Goding et al., 2014; Menendez and Alarcón, 2014; Menendez et al., 2014), a testable prediction would be that those cancers in which epigenome rewiring establishes a permissive milieu for carcinogenesis but requiring additional cooperating mutations for complete malignant transformation, should behave as accelerated models of oncogenesis. In contrast to sporadic forms, familial paragangliomas associated with mutations in the succinate dehydrogenase complex and the consequent accumulation of the histone demethylase (HDM) inhibitor succinate, which establishes a hypermethylator phenotype and the epigenetic silencing of key differentiation genes (Letouzé et al., 2013; Yang and Pollard, 2013), tend to present at a younger age (Lips et al., 2006; Chetty, 2010). Patients with gliomas with gain-of-function isocitrate dehydrogenase (IDH) mutations generating the HDM oncometabolite/inhibitor 2-hydroxyglutarate (2HG), which also establishes a hypermethylator phenotype that stabilizes undifferentiated cellular states that may be targetable and expanded later by subsequent transforming mutations, are, on average, several years younger that those with wild-type IDH gliomas (Bleeker et al., 2010; Cohen et al., 2013; Popov et al., 2013; Dimitrov et al., 2015). Moreover, we have recently identified how archetypal oncometabolites such as 2HG, by endowing cells with epigenetic states refractory to differentiation, considerably enhances the global kinetic efficiency of OSKM-driven nuclear reprogramming processes to generate cancer stem cell (CSC)-like states *de novo* (Menendez and Alarcón, 2016; Menendez et al., 2016). Altogether, these observations strongly support the notion that pathological versions of nuclear reprogramming could operate as primary and causative mechanisms of cancer-associated epigenetic rewiring.

### Environmental dedifferentiation of committed cells into stem cell-like states *in vivo*

There is accumulating robust evidence showing that non-stem cell compartments might be sources of newly generated pools of cells sharing stem-like characteristics with endogenous, adult stem cell counterparts in the same organ. The Weinberg group originally addressed this question to demonstrate that stem-like cellular states might arise *de novo* from more differentiated cell types within the human mammary gland (Chaffer et al., 2011). Moreover, a rare subpopulation of somatic cells of human breast tissue was found to be poised to actively transcribe plasticity and pluripotency markers such as *Oct4, Sox2*, and *Nanog*, and to acquire a plastic cell state sensitive to environmental programming (Roy et al., 2013).

Fully committed airway epithelial cells have been shown to revert to stable and functional stem cell-like states *in vivo* and, more importantly, to function as well as their endogenous adult stem cell counterparts in repairing epithelial injury (Tata et al., 2013). Upon crypt damage, Dll1^+^ intestinal secretory progenitor cells exhibit plasticity by regaining stemness (van Es et al., 2012). Additionally, the *Wnt* target gene leucine-rich-repeat containing G-protein-coupled receptor (*Lgr5*), which marks actively dividing stem cells in *Wnt*-driven, self-renewing tissues such as small intestine and colon, stomach and hair follicles (Barker et al., 2008), can be induced to form small Lgr5^+^ liver stem-like cells capable of generating hepatocytes and bile ducts *in vivo* (Huch et al., 2013). Upon damage, committed cells within tissues that have a low rate of spontaneous proliferation have the capacity to generate Lgr5^+^ stem cell-like states *de novo*, which are commonly observed in actively self-renewing tissues (Barker et al., 2008; Huch et al., 2013).

The demonstration that aberrant termination of OSKM-induced reprogramming *in vivo* results in tumor development also revealed the unexpected activation of *Lgr5* (Ohnishi et al., 2014a). Since *Lgr5* expression is not present in iPSCs, and is neither transiently expressed during reprogramming of fibroblasts nor in *in vitro* generated partially reprogrammed cells, its presence in OSKM-driven tumorigenesis raised important concerns regarding the relevance of this model as a proof-of-concept for epigenetics-driven cancer development *in vivo* (Hobbs and Polo, 2014; Ohnishi et al., 2014b). However, from a reparative/regenerative perspective, it is tempting to suggest that aberrantly terminated cellular reprogramming *in vivo* recapitulates the natural functioning of a host genetic program for resistance (e.g., regeneration of Lgr5^+^ stem-like cells from Lgr5^−^ cell populations) that is activated upon damage (Mosteiro et al., 2016).

## Aging and cancer: two sides of reparative cellular reprogramming

In close analogy to classical descriptions of nuclear reprogramming as a key regenerative mechanism in plants, invertebrates, teleost fishes, and amphibians (Brockes and Kumar, 2002; Jopling et al., 2011; Sugimoto et al., 2011), we are beginning to appreciate that the capacity of adult differentiated cells to generate transiently active stem-like cellular states challenges the commonly held belief that tissue-specific adult stem cells are the sole contributors to self-cell therapy (Desai and Krasnow, 2013). By functionally substituting tissue-specific stem cells, transiently reprogrammed mature committed cells might have a general role in adult tissue repair by operating in a host program for resistance to damage and other tissue adversities. *In vivo* reprogramming phenomena and consequent epigenetic plasticity, however, might also instigate tumor cell-like states by participating in the generation and maintenance of the versatility—aberrant differentiation and transdifferentiation capacities—of the CSC-like cellular states (Friedmann-Morvinski and Verma, 2014).

Cells with non-plastic chromatin will be less likely to undergo malignant transformation, but they will also be less able to respond to danger signals and, consequently, they will be more prone to degeneration. In this regard, the cyclic and transient expression of reprogramming factors *in vivo* has recently been shown to increase lifespan in a murine model of premature aging by remodeling the chromatin landscape (Ocampo et al., 2016). Conversely, cells with more plastic chromatin will be more adaptable in the face of cell intrinsic or microenvironmental changes, but they might also provide “molecular power” on a tissue's susceptibility to undergo aging-associated degeneration or cancer-associated malignant transformation. Accordingly, chronic injury and aging have been shown to render tissues highly permissive to *in vivo* reprogramming (Mosteiro et al., 2016). The goal now is to define the key players that are involved in regulating cellular plasticity, both during physiological *in vivo* reprogramming, leading to tissue rejuvenation, and during pathological conditions, where increased plasticity-related tissue dedifferentiation associates with cancer (Marión et al., 2017).

### Epigenetic plasticity and the archetypal pro-inflammatory cytokine IL-6

We are beginning to appreciate the existence of a common mechanism for epigenetic plasticity regulated by inflammatory signaling. However, while it is widely accepted that chronic inflammation may drive pathological changes in cell phenotypes, whether an inflammatory signal that is short-term and physiological can provide a molecular scenario capable of driving self-cell therapy for resistance to damage and disease remains a matter of discussion. The archetypal pro-inflammatory cytokine IL-6, which dictates the transition from acute to chronic inflammation, might illustrate how inflammation could have both a beneficial and a harmful role in aging and cancer (Scheller et al., 2011; Rincon, 2012; Hunter and Jones, 2015; Mauer et al., 2015).

Somatic cells can detect PAMPs or DAMPs *via* PRRs such as TLRs, the activation of which triggers the generation and release of chemokines and cytokines (e.g., IL-6) that contribute to inflammatory response (Kapetanovic et al., 2015; Toubai et al., 2016). Such an acute activation of inflammatory response provokes global changes in epigenetic modifiers, favoring an open chromatin configuration and increasing epigenetic plasticity. This temporary reprogramming would replenish damaged, diseased, and lost cells in tissues challenged with danger signals. The notion that inflammatory signaling might innately operate to boost the production of stem cell-like cellular states has been supported by the discovery that IL-6 plays an early yet critical role during generation of induced pluripotency. IL-6 is involved in reprogramming to pluripotency during embryogenesis (Zolti et al., 1991; Austgulen et al., 1995). Using non-dividing heterokaryons (murine ESCs fused to human fibroblasts) in which reprogramming toward pluripotency is efficient and rapid, the (undetectable) level of IL-6 in ESCs dramatically increases 50-fold upon during nuclear reprogramming (Brady et al., 2013). Moreover, exogenous addition of IL-6 can functionally replace the oncogenic *c-Myc* component of the Yamanaka cocktail during the generation of iPSCs (Brady et al., 2013).

At sites of transient inflammation, acute resolution of inflammatory response mediated by IL-6 (and downstream activation of NF-κB) could be accompanied by beneficial tissue regeneration (Cressman et al., 1996; Taub et al., 1999; Lasry and Ben-Neriah, 2015; Chiche et al., 2017), or reparative transdifferentiation (e.g., conversion of fibroblasts into endothelial cells to increase microvascular density in response to myocardial infarction-induced ischemic injury; Cooke et al., 2015; Sayed et al., 2015). At sites of chronic inflammation, if the culmination of transient reprogramming to stem-like epigenetic states is not accompanied by a committed re-acquisition of the original or alternative (but beneficial) differentiated cell fate, unrestrained nuclear reprogramming-driven tissue plasticity might impair the repair or replacement of damaged cells and at the same time generate cancer-like cellular states.

Chronic inflammation-related reduced regenerative capacity might be accompanied by permanent changes in target tissue cells involving either their locking in unresolved stem-like states or their transdifferentiation. Thus, while the IL-6-enriched microenvironment of the ≈20% of tumors that are associated with inflammation (e.g., chronic ulcerative colitis-associated colon cancer; Coussens and Werb, 2002; Grivennikov et al., 2010; Ben-Neriah and Karin, 2011; Balkwill and Mantovani, 2012) seems to have a dominant role in facilitating tumorigenesis with expansion and maintenance of CSC-like cells, chronic esophagitis caused by gastroesophageal reflux disease can promote transdifferentiation of stratified squamous esophageal epithelium into small intestine-like columnar epithelium (i.e., Barrett's esophagus), which might later be the site of malignant transformation (Kuilman et al., 2008; Vega et al., 2014; Kapoor et al., 2015; Wang and Souza, 2016). Furthermore, tumors that do not arise because of chronic inflammation appear to later develop an IL-6-rich microenvironment, which supports tumor progression and metastasis. Thus, an IL-6-driven inflammatory feedback loop is a core epigenetic regulator of the dynamic equilibrium that converts non-stem cancer cells into CSC-like cells, and generates tumor heterogeneity in genetically distinct cancer cells (Iliopoulos et al., 2011; Korkaya et al., 2011, 2012; Krishnamurthy et al., 2014). Indeed, IL-6/NF-κB-related signaling loops, which are recognized to lead to expansion of CSC-like populations, are reminiscent to those activated during chronic inflammation and wound healing, and provide a mechanistic basis for the known link between inflammation and the promotion of aggressive cancer phenotypes.

### Senescence-associated inflammatory signaling (SAIS): from organism-wide to local stress response and para-inflammation

The proposal that activation of *in vivo* cellular reprogramming is a host genetic program for resistance to damage and disease that could promote either “complete healing” of injured/diseased tissue or “incomplete healing” of old or cancerous tissues must be compatible with the accepted hypothesis that the degenerative and hyperplastic pathologies of aging, the most deadly of which is cancer, are linked by a common biological phenomenon: cellular senescence.

Cellular senescence is a persistent damage response in cells experiencing unresolved or irreparable stress for a sustained period of time. It has been causally linked to a wide variety of processes including wound healing, aging, tumor prevention, and tumor progression (Rodier and Campisi, 2011; Campisi, 2013; Tchkonia et al., 2013; Muñoz-Espín and Serrano, 2014; Lasry and Ben-Neriah, 2015). The multi-faceted capabilities of senescent cells involving both beneficial (tissue repair and tumor suppression) and deleterious (aging and tumor promotion) effects on organismal health can be viewed as consequences of senescence-associated inflammatory signaling (SAIS). Despite their loss of proliferative potential, senescent cells are metabolically and transcriptionally active and express a vast number of secreted proteins. With progressing age, an organism-wide increase of the senescence-associated secretory phenotype (SASP) ensues, which comprises a range of different proteins that are well-known players in aging and age-related diseases, including matrix-remodeling metalloproteases such as MMP3, growth factors such as HGF and TGFβ, inflammatory chemokines such as CCL2 and CLL11, and prominent pro-inflammatory cytokines such as IL1α/β, IL-6, and IL-8 (Coppé et al., 2008; Kuilman and Peeper, 2009; Orjalo et al., 2009; Rodier et al., 2009; Gross et al., 2012; Acosta et al., 2013). While the SASP of a cell varies according to tissue type and stressor, its ability to maintain not only the senescent cell itself but also to propagate the stress response and impact the microenvironment through communication with neighboring cells, can lead to organism-wide phenotypes *via* systemic inflammation that is largely dependent on the core inflammatory cytokines IL-6 and IL-8.

The senescence inflammatory response (SIR) is a second type of inflammation activated in senescent cells (Pribluda et al., 2013; Aran et al., 2016). Unlike the SASP, SIR is characterized by a small number of secreted factors and it is not accompanied by recruitment of immune cells into the senescent tissue. Intriguingly, SIR has a weak association with the NF-κB signaling pathway, but mostly comprises innate immunity proteins including members of the TLR activation pathway, which has an important role in tissue homeostasis by regulating inflammatory and tissue repair responses to injury (Rakoff-Nahoum and Medzhitov, 2009). Most likely, SIR has a largely epithelial-autonomous role and seems to contribute to tissue protective senescence and to counteract tumor progression by cooperating with *bona fide* tumor suppressor genes such as *p53* (Pribluda et al., 2013; Aran et al., 2016). Inflammatory processes such as transflammation and SIR might therefore be viewed as intermediate, tissue-level stress responses and para-inflammatory states, respectively, occurring between homeostasis and overt inflammation (Medzhitov, 2008; Chovatiya and Medzhitov, 2014). Accordingly, whereas transflammation is triggered by extrinsic assaults such as pathogens or tissue injuries, SIR seems to be prompted by tissue-intrinsic assaults such as DNA damage.

### SAIS: connecting the two sides of reparative cellular reprogramming

SAIS can be integrated with the continuum of the inflammatory spectrum that ranges from homeostatic states to (transflammation-like) stress responses (NF-κB-dependent and SIR-like), para-inflammation states, and finally overt (SASP-like) inflammation. Local stress and para-inflammation responses to extrinsic and intrinsic insults might arise first to respond to tissue stress-related danger signals (PAMPs and DAMPs) or to chronic tissue malfunction (DNA damage, senescence), later evolving into more systemic increases in the expression of major SASP factors, an organism-wide senescent phenotype that is accompanied by either immune clearance of senescent cells or attraction of inflammatory cells. Such multi-faceted, temporal organization of inflammatory phenotypes functionally converge into so-called “inflammaging,” defined as the low but chronic levels of inflammation associated with many protracted pathological conditions (e.g., atherosclerosis, diabetes mellitus, autoimmune diseases, and neurodegeneration), which are thought to drive age-related decline in function (Franceschi and Campisi, 2014). The causal role of SAIS as a pivotal driver of the age-related decline in tissue homeostasis is evidenced by: (a) increased expression of genes linked to immune responses and inflammation in aging tissues; (b) chronic activation of NF-κB; (c) organism-wide elevated levels of major SASP factors such as IL-6 in a number of models of physiological and accelerated aging (Baker et al., 2008; Gregg et al., 2012; Wiley et al., 2016); and (d) a prolonged healthspan and extension in lifespan upon semigenetic clearance or pharmacological/directed elimination of senescent cells with so-called senolytic drugs (Baker et al., 2008, 2011, 2016; Zhu et al., 2015; Chang et al., 2016; Yosef et al., 2016).

**Figure 2:**
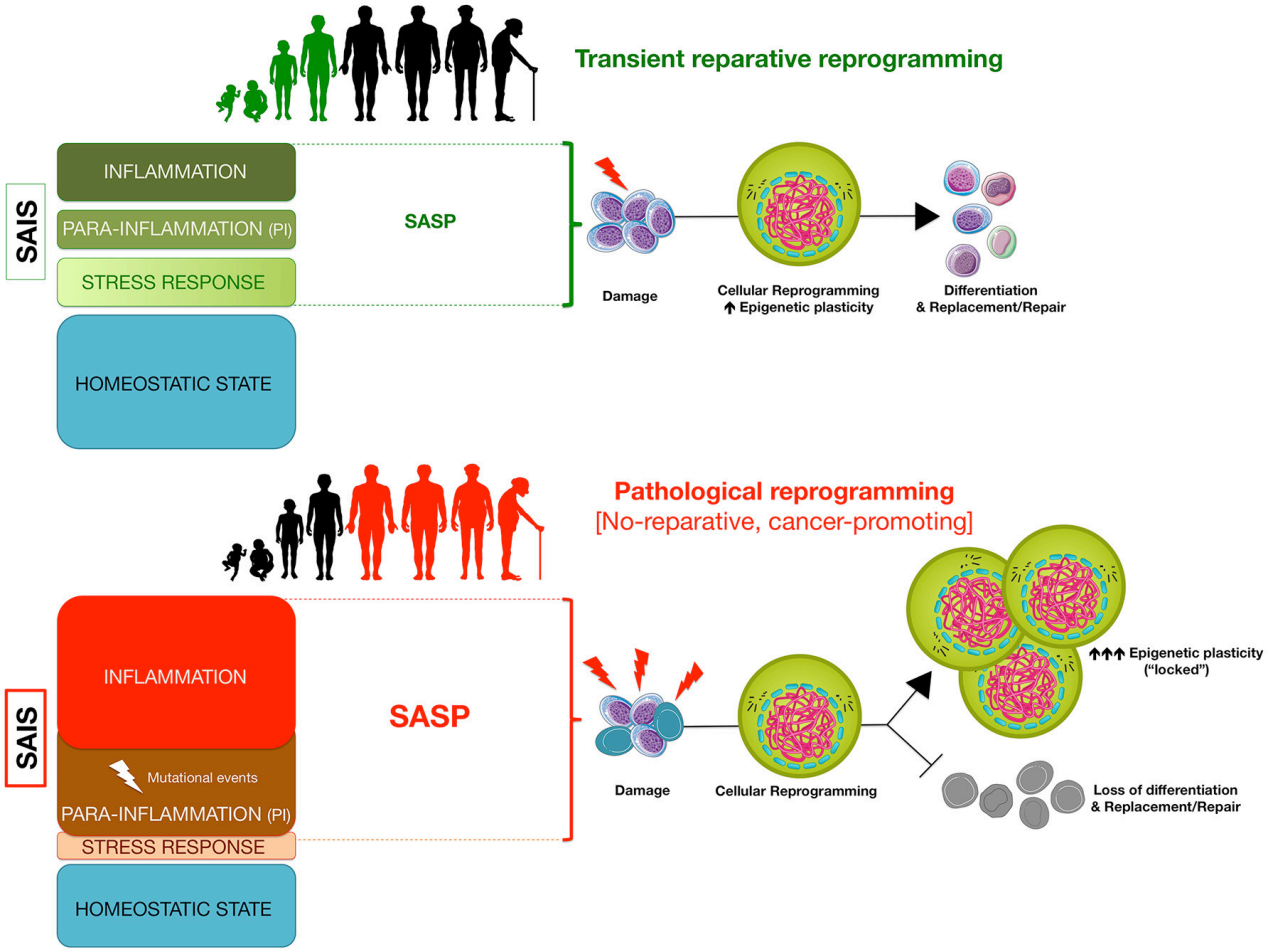
**Senescence-associated inflammatory signaling (SAIS)-regulated ***in vivo*** reprogramming: a threshold model of epigenetic plasticity in aging and cancer**. The degree of senescence/inflammation deviation from the homeostatic state delineates a thresholding algorithm distinguishing beneficial vs. deleterious effects of *in vivo* reprogramming. First, transient activation of innate immunity and/or SASP components (e.g., IL-6) might facilitate reparative cellular reprogramming in response to acute inflammatory events. Second, NFκB-dependent and NFκB-independent (e.g., SIR) para-inflammation switches might promote a long-lasting but reversible refractoriness to reparative cellular reprogramming. Third, chronic SASP might lock cells into highly plastic epigenetic states disabled for reparative differentiation capacities.

The temporal continuum of SAIS closely relates to the well-recognized antagonistic pleiotropy of senescence (Campisi, 2005). Senescence can be beneficial early in life or under transient conditions of injury. For instance, senescence promotes correct patterning of the embryo (Muñoz-Espín et al., 2013; Storer et al., 2013) and, after development, SAIS can be beneficial by aiding in wound healing and limiting fibrosis following acute damage (Krizhanovsky et al., 2008; Demaria et al., 2014). However, it is later in life when SAIS, after crossing arbitrary thresholds at both the cell-autonomous and non-cell autonomous levels, is thought to be responsible for many aging-related disorders. On the assumption that terminally differentiated cells can transiently regain core stem cell-like functional properties, one could hypothesize that the beneficial or deleterious paths ensuing upon cellular reprogramming-like cycles of tissue maintenance might be dictated by cell-autonomous and non-cell autonomous SAIS capable of making tissue cells not only transiently exceed “reprogramming barriers,” but also to re-acquire the original or alternative differentiated cell fate, in a difficult or easier manner. The degree of deviation from the homeostatic state might establish biological constraints delineating a multiple thresholding algorithm that isolates zones of beneficial vs. deleterious SAIS-regulated reparative cellular reprogramming (Figure 2).

#### Transflammation: innate immunity-facilitated reparative cellular reprogramming

From a cell-autonomous perspective, transflammation involving endogenous activation of innate immunity to PAMPs or DAMPs might be sufficient to modify the expression or activity of epigenetic modifiers to generate phenotypic fluidity for cellular responses to pathogenesis or injury (Cooke et al., 2014). If acutely resolved, such a temporal stress response-inflammatory process might allow temporary cell reprogramming and re-acquisition of the original or alternative cell fate in response to specific environmental cues, leading to beneficial tissue rejuvenation or transdifferentiation, respectively. Transflammation-driven reparative cellular reprogramming, which is expected to mostly involve increases in epigenetic plasticity to allow functional malleability without the loss in cellular identity, might operate as a *bona fide* protective response to challenge and eliminate pathogens and also to biophysical tissue damage.

The above scenario, although lacking rigorous experimental validation, is strongly supported by the recent uncoupling of rejuvenation from dedifferentiation associated with OSKM-driven reprogramming of somatic cells. First, brief exposure to OSKM factors, allowing cells to transiently transition through a plastic intermediate state without the complete loss of cellular identity, allows indirect lineage conversion of human fibroblasts to angioblast-like cells with reparative potential in ischemic pathologies (Kurian et al., 2013). Second, at the organism level, the cyclic and short-term expression of OSKM factors can transiently revert premature aging phenotypes including DNA damage responses and senescence-associated features without involving the complete loss of cellular identity (Ocampo et al., 2016).

#### Para-inflammation switches: loss of cellular resilience without tissue repair and rejuvenation

Since unrestricted changes in cell identity may also predispose to loss of tissue homeostasis, it is reasonable to suggest that the optimal zone for innate immunity-facilitated cellular reprogramming to operate as a reparative mechanism will be small. Whereas the lower threshold is expected to be greater than the baseline inflammatory value arising from disturbed/perturbed physiological homeostasis, a short arbitrary “inflammatory distance” to the upper threshold should ensure that protective inflammatory repair processes might become pathogenic by altering the epigenetic states of damaged/diseased tissue cells. The narrow nature of the optimal zone for transflammation-driven reparative cellular reprogramming should have two major consequences. First, exaggerated or uncontrolled responses to PAMPs and DAMPs resulting in acute systemic hyperinflammation or repeated, overshooting of repair will overcome the upper threshold of the optimal zone of innate immunity-facilitated reparative epigenetic plasticity, thus impeding restoration of tissue homeostasis but eliciting collateral tissue damage (e.g., fibrosis). Secondly, the inability to generate new pools of stem-like cells, for instance, due to chronic baseline inflammatory scenarios exceeding the upper threshold, might hamper a crucial source of self-repair.

An unresolved scenario of continuous cell stress/tissue injury accompanied by inappropriate resolution of endogenous (e.g., metabolic and genomic damage) or exogenous (e.g., pathogens, biophysical stresses) assaults will associate with the activation of senescence-associated para-inflammatory states. The baseline SAIS level of such states will exceed the upper inflammation threshold and will be characterized by a progressive loss of cellular resilience that, however, will not be accompanied by rejuvenation-like phenomena. These para-inflammatory states, which can originate in a NF-κB-independent cell-autonomous manner (e.g., SIR) or in a more systemic NF-κB-dependent manner (Soria-Valles et al., 2016), would operate as senescence-inflammatory friend-or-foe switches that, while originally contributing to tissue protective senescence and counteracting tumor progression, can also drive reprogramming-refractory aging phenotypes and cancer-prone epithelial tissue (Figure 2).

Some testable predictions of the switching nature of SAIS arising from para-inflammatory states include: (a) the initial ability of para-inflammation SAIS to suppress the potential of stressed cells acquiring a malignant state will be lost in response to certain environmental and genetic clues (e.g., loss of tumor suppressor genes); and (b) the reduction in SAIS might force such para-inflammatory aging and malignant states to return to an optimal zone of transient reprogramming for rejuvenation while depleting cancer aggressiveness. Accordingly, aberrant NFκB activation is known to impair somatic cell reprogramming and to drive the aging phenotype while also promoting the expansion of CSCs *via* cell- and non-cell autonomous mechanisms (Colotta et al., 2009; Shostak and Chariot, 2011; Yamamoto et al., 2013; Terlizzi et al., 2014; Soria-Valles et al., 2016). NFκB inhibition, which is a potential therapeutic strategy to eliminate CSCs, has been shown to delay DNA damage-induced senescence and aging in mice and to significantly increase the reprogramming efficiency of fibroblasts from patients with progeria syndrome as well as from normal aged individuals (Tilstra et al., 2012; Soria-Valles et al., 2015a,b). These findings lend weight to the notion that targeting NFκB-related SAIS might modify the thresholds for reparative cellular reprogramming. Moreover, whereas para-inflammation-associated SIR is widely prevalent in cancers harboring mutations in *p53* (Aran et al., 2016), the capacity for non-steroidal anti-inflammatory drugs (NSAIDs) such as aspirin to exert protective effects against several cancers (e.g., colorectal, pancreatic, lung, and breast; Rothwell et al., 2011; Fraser et al., 2014; Streicher et al., 2014) might be related to their ability to suppress key drivers of SIR in poor prognosis, para-inflammated tumors (Aran et al., 2016). Interestingly, NSAIDs can enhance cellular reprogramming even in the absence of *Sox2* and *c-Myc* (Yang et al., 2011), thus providing further evidence for the thresholding capacity of inflammation-epigenetic axes to determine the optimal zones of cellular reprogramming-driven phenotypic plasticity.

#### Chronic SASP and loss of tissue homeostasis: the “stem-lock” zone

From a non-cell autonomous perspective, if the loss of differentiation features following reprogramming is not accompanied by re-acquisition of the original or alternative differentiated cell fate, the resulting tissue plasticity might impair the repair or replacement of damaged cells. The ability of SASP-associated pro-inflammatory cytokines to regulate stemness and nuclear reprogramming raises the notion that a SASP-impaired local environment could interfere with tissue rejuvenation by imposing the so-called “stem-lock” state (de Keizer, 2017). Chronic inflammatory conditions *via* exposure to IL-1, which normally functions as a key “emergency” signal and a master regulator of SASP by inducing downstream effectors such as IL-6, has been shown to impair tissue homeostasis and to induce an aged appearance of the hematopoietic system by restricting stem cell differentiation (Pietras et al., 2016). Moreover, biological conditions linked to chronic senescence, such as tissue injury or aging, favor *in vivo* OSKM-driven reprogramming *via* enhanced production of IL-6 as shown by the appearance of *Nanog*-positive cells in the vicinity of senescence areas (Mosteiro et al., 2016).

While counterintuitive, the ability of SASP factors including IL-6 to transiently create a permissive environment for *in vivo* reprogramming capable of inducing cellular plasticity and tissue regeneration (Ritschka et al., 2017), a prolonged promotion of such progenerative response might reduce tissue rejuvenation and promote aging by self-enhancing futile cycles of SASP/IL-6-driven reparative cellular reprogramming. Compared with young tissues containing few senescent cells where transient SAIS might cause temporary reprogramming and differentiation/proliferation to replenish cells, the prolonged accumulation of senescent cells in tissues that are old or under high levels of stress (e.g., following medical procedures such as chemotherapy) might be accompanied by a defective clearance of damaged, senescent cells, which can promote further SASP accumulation. A situation of chronic SASP secretion might not only counter the continued regenerative stimuli by promoting cell-intrinsic senescence arrest in single damaged cells but also paradoxically impose a permanent, locked gain of stem cell-like cellular states with blocked differentiation capabilities in surrounding cells (Figure 2). Such a scenario of prolonged survival of senescent cells and enhanced phenotypic plasticity of neighboring cells would drive a loss of tissue homeostasis by impeding the reparative replenishment of damaged cells. As mentioned earlier, core SASP factors such as IL-6 can mimic the effects of *in vivo* reprogramming (Mosteiro et al., 2016), thereby favoring the emergence of CSC-like cellular states in neighboring cancer cells (Cahu et al., 2012; Chang et al., 2015). Thus, chronic SASP-driven loss of tissue homeostasis might go hand-in-hand with an accelerated generation of trade-off forms of undifferentiated types of cells with CSC-like states. Accordingly, the protracted presence of senescent cells that can promote local and systemic SASP in stressed normal tissue has recently been shown to cause and exacerbate short- and long-term effects of genotoxic stresses ranging from weakness and fatigue in skeletal muscle to CSC-related cancer recurrence (Demaria et al., 2017).

## Senescence-inflammatory regulation of reparative cellular reprogramming in aging and cancer: A threshold model of epigenetic plasticity

Nuclear reprogramming-like phenomena inducing transient epigenetic plasticity followed by cell differentiation and replacement of damaged/diseased cells may constitute a previously unrecognized route through which human tissue responds to injury, stress, and disease. This may lead to either acutely resolved tissue repair (e.g., transient gain of epigenetic plasticity upon acute transflammation events) or, alternatively, to undesirable, chronically unresolved tissue damage (e.g., lasting gain of epigenetic plasticity upon chronic SASP responses). Aging and cancer might thus be viewed as the consequence of an unrestricted/unresolved stimulation of futile, non-reparative *in vivo* reprogramming-driven epigenetic plasticity in response to chronic, but reversible, senescence-inflammatory signaling.

**Figure 3:**
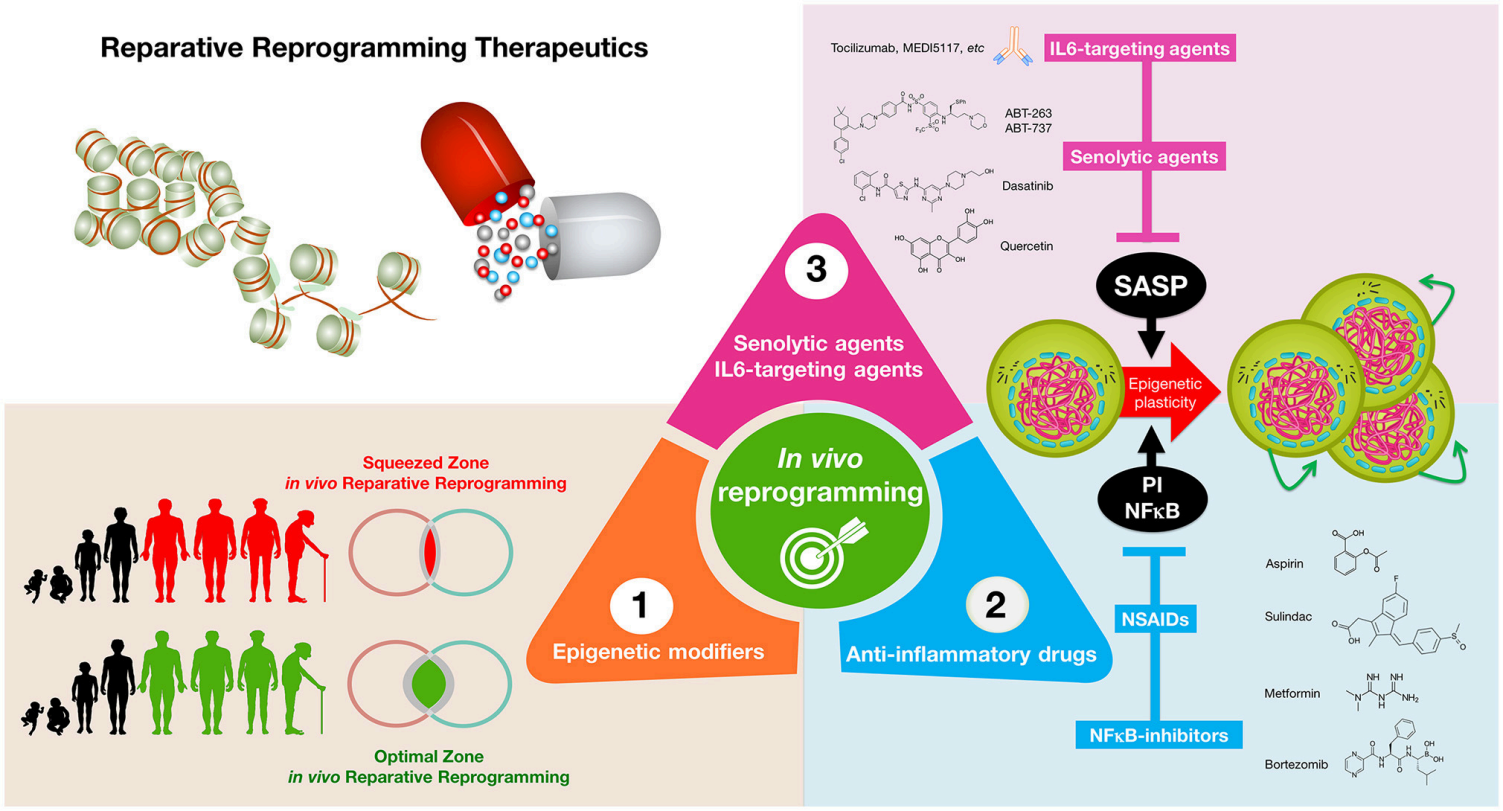
**Reparative reprogramming therapeutics: enhancing the body's self-cell therapy for resistance to damage and disease**. A cellular reprogramming-centered view of epigenetic plasticity as a fundamental dimension of a tissue's capacity to undergo successful repair may provide new therapeutic approaches for aging and cancer. (1) *Epigenetic modifiers:* small molecules capable of mimicking the transient amelioration of tissue functions occurring upon short-term induction of OSKM-induced nuclear reprogramming (Mahmoudi and Brunet, 2016; Ocampo et al., 2016) might increase epigenetic plasticity and to enhance regeneration in aging tissues; (2) *anti-inflammatory drugs:* NFκB-targeting drugs and commonly employed NSAIDs might help reduce some aging- and cancer-promoting inflammatory feedback loops to reestablish the functioning of reparative reprogramming; (3) *IL-6-targeting and senolytic agents:* IL-6 blockade and senescent cell ablation might help unlock the chronic epigenetic plasticity of SASP-damaged tissues to successfully achieve tissue rejuvenation if accompanied by reparative differentiation phenomena.

We propose that the regulation of cellular reprogramming/differentiation cycles of tissue repair by the cell-autonomous/non-cell autonomous mechanisms that initiate and propagate SAIS might suffice to outline a threshold model of epigenetic plasticity in aging and cancer. A better understanding of the biological constraints that determine how the map of SAIS-regulated reparative *in vivo* reprogramming is dynamically thresholded may provide therapeutic approaches for aging and cancer (Figure 3). A first open question is whether “epigenetic bursts” of innate immunity-facilitated reparative epigenetic plasticity operate as physiological mimickers of the transient amelioration of tissue functions without inducing complete dedifferentiation, as apparently occurs upon short-term induction of OSKM factors in animal models (Mahmoudi and Brunet, 2016; Ocampo et al., 2016). In such a scenario, the discovery and validation of small molecules, more likely epigenetic modifiers, capable of widening or re-establishing the optimal zone of physiological *in vivo* reprogramming would be expected to increase epigenetic plasticity and to enhance regeneration in aging tissues. A second open question is whether commonly employed NSAIDs (e.g., aspirin, sulindac derivatives; Chan and Detering, 2013; Gurpinar et al., 2014) and NFκB-targeting drugs (e.g., bortezomib, metformin; Hirsch et al., 2013; Zhou et al., 2015) can reestablish stress response-inflammatory thresholds compatible with reparative reprogramming while eliminating aging- and cancer-promoting inflammatory feedback loops. Finally, a third open question concerns the clarification of how senescent cells operate as *bona fide* sources of *in vivo* reprogramming. The discovery of the first generation of senolytic drugs (Kirkland and Tchkonia, 2015; Zhu et al., 2015, 2016; Chang et al., 2016) along with therapeutics targeting core SASP components such as IL-6 (Krishnamurthy et al., 2014; Kim et al., 2015; Heo et al., 2016; Zhong et al., 2016) might be viewed as an obvious therapeutic avenue to stimulate *in vivo* reprogramming-driven tissue rejuvenation. However, it should be acknowledged that an ideal anti-aging therapy would need not only to “unlock” the chronic epigenetic plasticity of SASP-damaged tissues, but also to stimulate differentiation of stem cell-like states to successfully achieve tissue rejuvenation (de Keizer, 2017). Nonetheless, it would be interesting to evaluate whether, beyond IL-6 blockade, senescent cell ablation might also ameliorate the efficacy of cancer treatment modalities by impeding the replenishment of treatment-refractory CSCs that might *de novo* arise by cellular reprogramming-like phenomena of non-CSC tumor counterparts.

The consideration of a cellular reprogramming-centered view of epigenetic plasticity as a fundamental element of a tissue's capacity to undergo successful repair, aging degeneration or malignant transformation should provide stochastic insights into the current deterministic genetic paradigm for aging-related diseases, thereby increasing the spectrum of therapeutic approaches for physiological aging and cancer.

## Author contributions

JM and TA conceived the idea for this project and wrote the manuscript.

### Conflict of interest statement

The authors declare that the research was conducted in the absence of any commercial or financial relationships that could be construed as a potential conflict of interest.
